# Lymphatic dysfunction correlates with inflammation in a mouse model of amyotrophic lateral sclerosis

**DOI:** 10.1242/dmm.052148

**Published:** 2025-07-16

**Authors:** Akshaya Narayanan, Bonnie L. Seaberg, Andrew Buxton, Alexandra Vernino, Victoria E. Williams, Anthony Matarazzo, Jeet Kekre, Bhuvaneshwaran Subramanian, Wei Wang, Joseph M. Rutkowski, Michelle Hook, Dylan A. McCreedy, Mariappan Muthuchamy, Mendell Rimer

**Affiliations:** ^1^ Department of Medical Physiology, Texas A&M University, College Station, TX 77843, USA; ^2^ Department of Neuroscience and Experimental Therapeutics, Texas A&M University, College Station, TX 77843, USA; ^3^ Department of Biomedical Engineering, Texas A&M University, College Station, TX 77843, USA; ^4^ Department of Biology, Texas A&M University, College Station, TX 77843, USA

**Keywords:** Lymphatics, Lymphangiogenesis, Amyotrophic lateral sclerosis, Systemic inflammation, Neuroinflammation

## Abstract

Amyotrophic lateral sclerosis (ALS) is a rapidly progressive, ultimately fatal neurodegenerative disease, without effective modifying treatments. It affects both lower and upper motor neurons, causing skeletal muscle denervation and paralysis. Regardless of the mechanisms that initiate and drive ALS, chronic neuroinflammation and systemic immune system activation play key roles in disease progression. The lymphatic system is a network of vessels and organs essential for immune surveillance, tissue fluid balance and lipid absorption, critical for the resolution and progression of inflammation in the periphery. Its recent rediscovery in the central nervous system raises the possibility of it playing similar roles in neurological and neurodegenerative diseases featuring prominent neuroinflammation, such as ALS. We hypothesized that the structure and function of lymphatics are compromised in the most widely used murine model of ALS, the SOD1-G93A mouse. We found that these mice exhibit lymph transport dysfunction, diminished intrinsic lymphatic vessel tonic and phasic contractions, and an association between inflammation and lymphatic marker upregulation, despite absence of major structural changes in lymphatic network coverage in key affected tissues in the disease, skeletal muscle and spinal cord.

## Teaser

**Summary:** This is the first characterization of the structure and function of the lymphatic system in the SOD1-G93A mouse model of amyotrophic lateral sclerosis.

## INTRODUCTION

Amyotrophic lateral sclerosis (ALS) is a rapidly progressive, ultimately fatal neurodegenerative disease that affects both lower and upper motor neurons. Although heterogeneous in terms of site, age of onset, rate of progression and survival, most cases present clinically in late middle age with localized bulbar or limb muscle weakness that quickly turns into muscle atrophy, denervation and paralysis in most of the body. Death due to respiratory failure ensues 3-5 years after initial diagnosis. While >90% cases have an unknown cause (i.e. are sporadic), ∼5-10% of cases arise as a result of – mostly – gain-of-function, dominant mutations in over 40 genes, the most frequent of which are *C9orf72*, *TARDBP*, *FUS*, *SOD1* and *TBK1* (Gregory et al., 2020)*.* ALS currently lacks effective disease-modifying treatments, highlighting the urgent need for new interventions that significantly reduce patient functional decline, increase survival and improve quality of life (Barp et al., 2020; Masrori and Van Damme, 2020).

Although precise causes for ALS remain unknown, it is clear that multiple, complex cellular mechanisms go awry in motor neurons, glial and skeletal muscle cells, which together contribute to the loss of neuromuscular synaptic connectivity, axonal degeneration, muscle atrophy and motor neuron death typical of ALS. Regardless of the mechanisms that initiate and drive ALS, excessive neuroinflammation – characterized by local astrocyte and microglial activation, T-cell infiltration and systemic immune system activation – plays a key role in disease progression (Beers and Appel, 2019; Appel et al., 2021; Burberry et al., 2020; Beland et al., 2020; McCauley and Baloh, 2019; McCombe et al., 2020; Chiu et al., 2009).

The lymphatic system that runs in parallel with the blood vascular system is responsible for the daily absorption of 20-50% of the plasma volume and 50-100% of the plasma proteins from the interstitium, draining them back into the systemic circulation. In addition, lymphatic vessels function as active conduits for the passage of extravasated leukocytes and immune cells such as antigen-presenting dendritic cells, T lymphocytes and macrophages, thus representing an important component in the regulation of the immune response (Vranova and Halin, 2014)*.* In peripheral tissues, lymphatic vessels start as blind-ended, thin capillaries that then merge into afferent collecting vessels that drain into lymphatic nodes. From lymphatic nodes, fluid and cells (i.e. lymph) are returned to the circulation through efferent collecting lymphatics (Vranova and Halin, 2014; Oliver et al., 2020). Both initial and collecting lymphatics consist of lymphatic endothelial cells (LECs) joined together by either discontinuous, button-like junctions that facilitate fluid and cellular uptake (initial lymphatics) or by tighter, continuous zipper-like junctions (collecting lymphatics) (Oliver et al., 2020). Collecting lymphatics are both covered by specialized, contractile lymphatic muscle cells that aid in lymph flow and have valves that ensure unidirectional lymph movement (Muthuchamy et al., 2003; Oliver et al., 2020; Vranova and Halin, 2014). Skeletal muscle contraction and arterial wall pulsation are also thought to assist in driving lymph flow (Alitalo, 2011). Lymphatic vessels have been found in most vascularized tissues, including skeletal muscle; but, in the brain and spinal cord (SPC), they have been recently localized not to the parenchyma but to the meninges, three overlapping membranes that envelope and protect the central nervous system (CNS). Thus, lymphatic vessels in the CNS are referred to as meningeal lymphatics, more specifically as dural lymphatics, because most of them are found in this outermost meningeal layer (Oliver et al., 2020; Louveau et al., 2015; Aspelund et al., 2015; Jacob et al., 2019).

Vascular endothelial growth factor (VEGF)-C and VEGF-D, and their cognate receptor VEGFR-3 (also known as FLT4) are key for the LEC sprouting, proliferation and migration that are necessary for the generation of lymphatic vessels (i.e. lymphangiogenesis) (Alitalo, 2011; Oliver et al., 2020). An inflammatory environment induces gene expression changes in LECs that, in turn, lead to expansion of the lymphatic network via lymphangiogenesis as an adaptive response that can be beneficial or harmful, depending on the specific disease context (Kim et al., 2014; Abouelkheir et al., 2017). Experimentally enhancing or inhibiting lymphangiogenesis via manipulation of the VEGF-C/D–VEGFR-3 signaling pathway has been shown to modulate inflammation and immune responses in several disease models (D'Alessio et al., 2014; Dietrich et al., 2010; Huggenberger et al., 2010; Lopez Gelston et al., 2018). This raises the exciting possibility of harnessing the lymphatic system as a therapeutic target in multiple inflammatory diseases.

Given the prominent role of neuroinflammation and peripheral inflammation in ALS pathology progression (Appel et al., 2021; Beland et al., 2020; McCombe et al., 2020), we hypothesize that lymphatic structure and function are compromised in the most widely used animal model of ALS, the SOD1-G93A mouse (Gurney et al., 1994).

## RESULTS

We first assessed body weight, forelimb grip strength, hindlimb tremor and splay to determine disease progression in the SOD1-G93A animals in our hands. This characterization focused on male mice. Unless otherwise indicated, male mice were used in our experiments because of their previously reported earlier disease onset, more severe neuromuscular pathology and more pronounced neuroinflammation relative to those of females (Naumenko et al., 2011; Shelest et al., 2024 preprint; Pfohl et al., 2015). Consistent with previous reports (Mead et al., 2011; Lee et al., 2013), the overt clinical symptoms we studied developed approximately around postnatal day (p)75 and continued worsening thereafter until reaching end stage at ∼p160 (Fig. S1). Based on these data, we defined the clinically presymptomatic stage occurring prior to p75 and the clinically postsymptomatic stage after p75. Henceforth, presymptomatic stage experiments were carried out with p50 mice, whereas postsymptomatic stage experiments were done with p120-p126 mice.

### SOD1-G93A ALS mice display a decrease in lymph transport

**Fig. 1.**
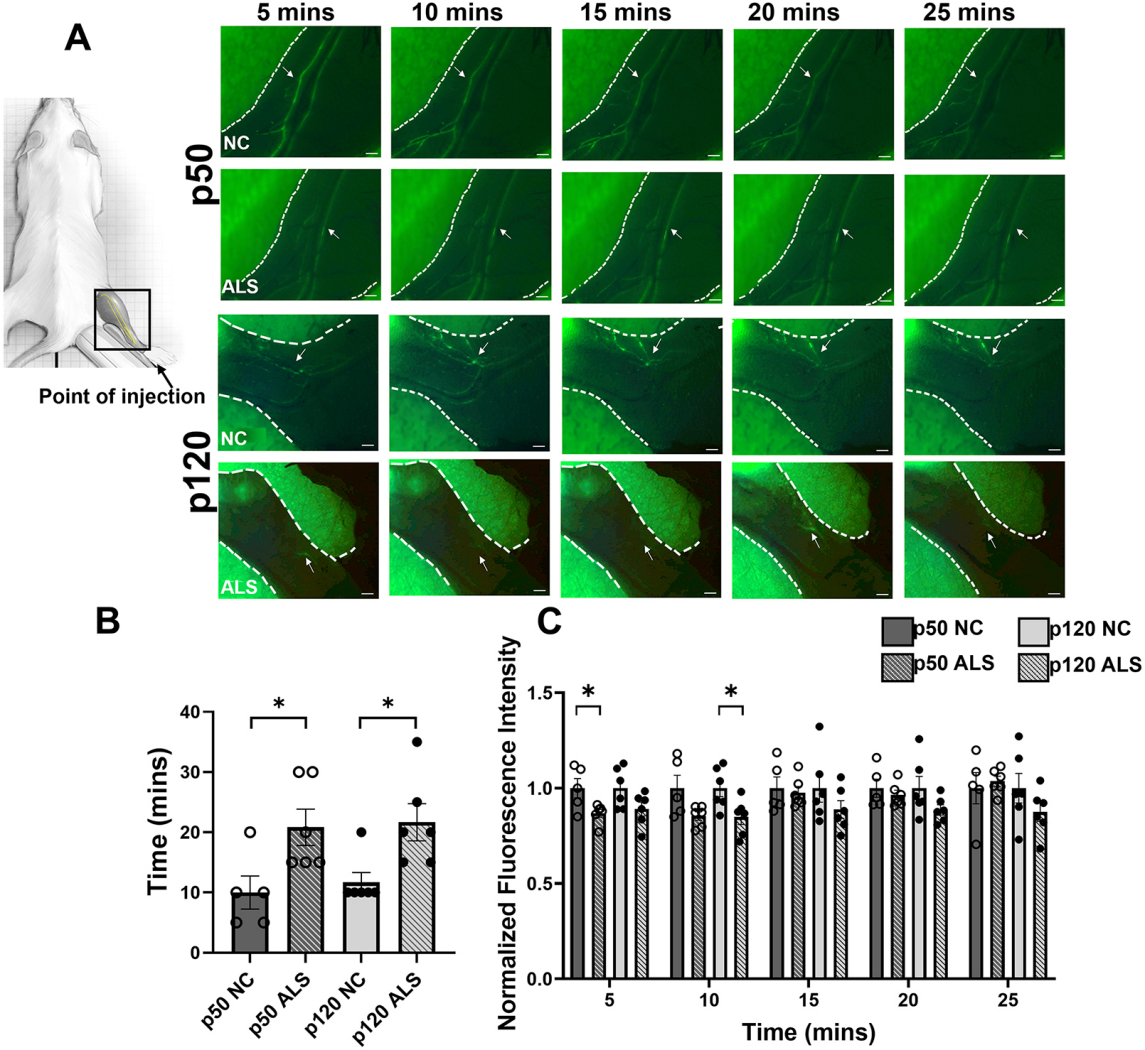
**Lymph transport is delayed in the lower limbs of presymptomatic and postsymptomatic SOD1-G93A ALS mice**. (A) Cartoon to the left shows the site of injection of FITC-Dextran in the shaved right leg paw and the area in the right hindlimb monitored under a fluorescent scope after that (boxed). The right panel shows time-lapse sequential images taken every 5 min after FITC-Dextran injection, showing the peripheral lymphatics (arrows) in the right hindlimb (outlined within dashed lines) of postnatal day (p)50 and p120 non-transgene-carrier (NC) control and amyotrophic lateral sclerosis (ALS) SOD1-G93A mice. (B) Average time taken to attain maximum fluorescence intensity, which was longer on average for the ALS mice, was measured and plotted for control and ALS groups. (C) Average fluorescence intensity in a standardized area was quantified in ImageJ at each time point up to 25 min for control and ALS groups. *n*=6/genotype/disease stage, all males. NC animals of the same age were used as controls for p50 and p120 ALS mice, respectively. *P<0.05. Values are presented as mean±s.e.m. Means were compared using Mann–Whitney test. Scale bars: 410 µm.

To determine *in vivo* lymphatic vessel function in ALS mice, we employed fluorescence microlymphangiography and quantified lymph transport in the lower limbs of age- and sex-matched control [non-transgene-carrier (NC)], clinically presymptomatic (p50) and postsymptomatic (p120) ALS mice. A representative panel of images (Fig. 1A) shows the transport of FITC-Dextran by the peripheral lower-limb lymphatic vessels in NC and ALS mice at both stages. As seen in Fig. 1B, the time for the popliteal vessel to attain maximum fluorescence intensity was significantly longer in the ALS mice compared to that in the control mice at both p50 and p120 (p50: 20.8±3.004 min for ALS vs 10.0±2.74 min for control, *P*=0.048; p120: 21.6±3.07 mins for ALS vs 11.66±1.67 mins for control, *P*=0.011), indicative of reduced macromolecule uptake rate. Additionally, the amount of lymph carried, measured by the fluorescent intensity at a given time, tended to be reduced compared to that for their respective controls (Fig. 1C) at both p50 and p120. Thus, these data demonstrate that lymph transport function is significantly reduced in lower limbs of the ALS mice, both at the presymptomatic and postsymptomatic stages of the disease.

### Lymphatic vessel contractile activity is impaired in SOD1-G93A ALS mice

**Fig. 2.**
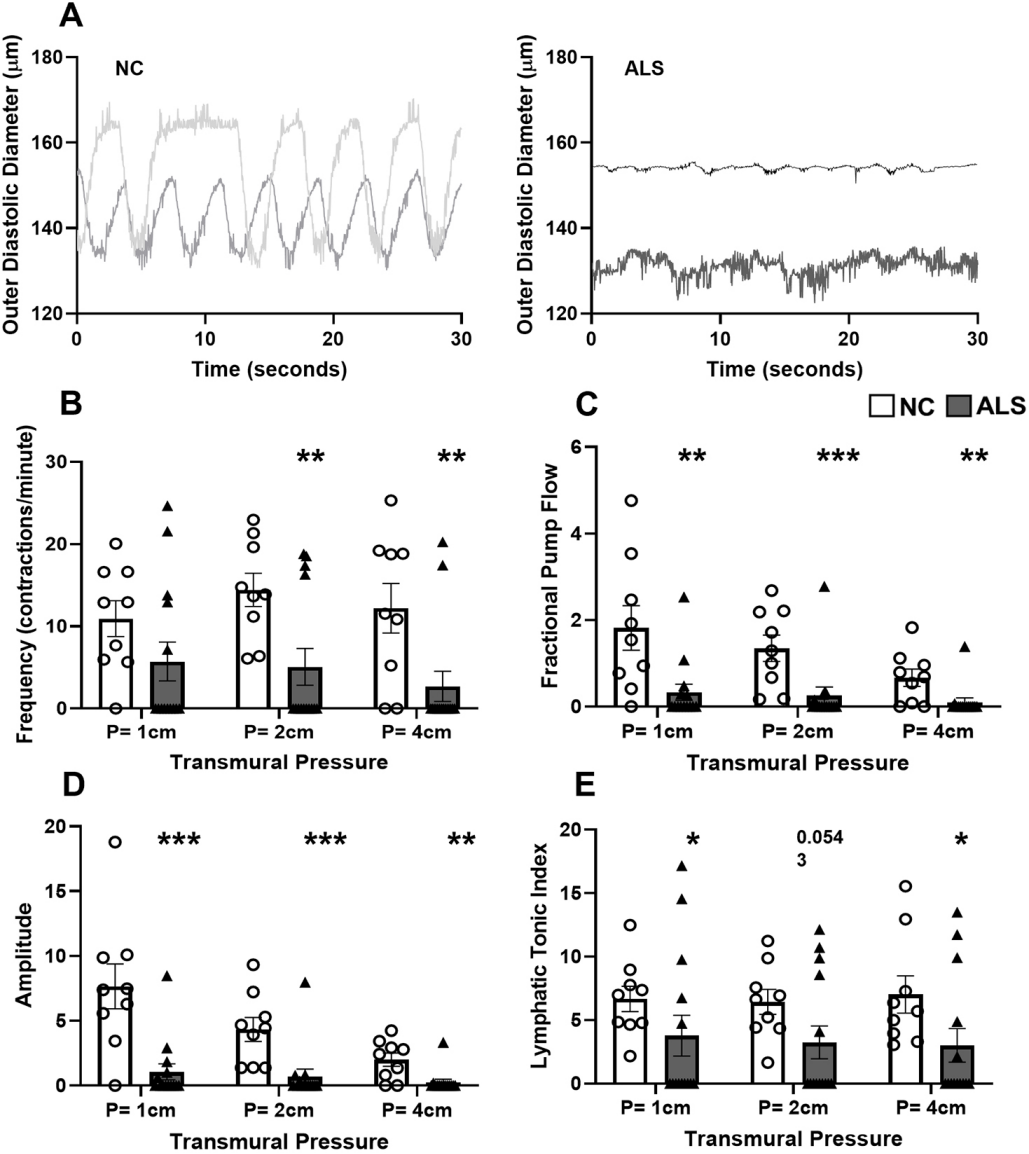
**Lymphatic vessel contractile activity is impaired in postsymptomatic SOD1-G93A ALS mice.** (A) Representative diameter traces over 30 s period in NC control and ALS lymphatic vessels at a transmural pressure of 1 cm. (B) Phasic contractile frequency. (C-E) Fractional pump flow (C), amplitude (D) and lymphatic tonic index (E) were calculated as described in the Materials and Methods. For NC, *N*=9 vessels from four animals; for ALS, *N*=14 vessels from four animals. *P<0.05, **P<0.01, ***P<0.001. Values are presented as mean±s.e.m. Means were compared using Mann–Whitney test.

To further determine whether the collecting lymphatic contractile activity is compromised in the ALS mice, isolated lymphatic vessel studies, from p120 postsymptomatic mice, were performed. Fig. 2A shows representative diameter traces of pressurized flank lymphatic vessels obtained from two NC and two ALS mice. In the NC mice, all nine isolated lymphatic vessels examined exhibited phasic contractile activity, whereas in ALS mice, only five of 14 vessels examined showed phasic contractile activity at all pressures tested. Additionally, the contractions that did occur in ALS mice were weak and pulsatile in nature compared to the stronger phasic contractions observed in the NC mice. A summary of lymphatic vessels diastolic and systolic diameters and calculated contractile parameter values for frequency, fractional pump flow, ejection fraction, amplitude and tone index are provided in Table 1. Although the phasic contractile frequency was not significantly decreased at 1 cmH_2_O pressure, it was significantly decreased at 2 and 4 cmH_2_0 pressures in the lymphatic vessels from ALS mice compared to the control mouse vessels (Table 1 and Fig. 2B). Additionally, the fractional pump flow, which is the product of phasic contractile frequency and ejection fraction, and the phasic contraction amplitude were significantly diminished at all the pressures tested in the vessels isolated from ALS mice (Fig. 2C,D and Table 1). The lymphatic tonic index values were also significantly different between the control and ALS groups (Fig. 2E and Table 1). Thus, these data demonstrate that the lymphatic tonic and phasic contractile activities, contractile frequency and amplitude are significantly reduced in the ALS mouse flank lymphatic vessels.

**Table 1.** Contractile parameters of isolated flank lymphatics from postnatal day 120 NC and ALS mice*

Parameters	Cohort	P=1 cmH_2_0	*P*-value	P=2 cmH_2_0	*P*-value	P=4 cmH_2_0	*P*-value
Frequency per min	NC	10.94±2.169	0.0964	14.43±2.018	0.0087	12.20±3.021	0.0056
	ALS	5.723±2.371		5.074±2.230		2.692±1.835	
Fractional pump flow (min^−1^)	NC	1.817±0.517	0.0033	1.346±0.305	0.0005	0.6677±0.201	0.0019
	ALS	0.3318±0.188		0.2534±0.197		0.1041±0.099	
Ejection fraction	NC	0.1549±0.034	0.0008	0.0906±0.019	<0.0001	0.0422±0.011	0.0019
	ALS	0.0227±0.013		0.0152±0.012		0.0056±0.005	
Contraction amplitude (%)	NC	7.659±1.738	0.0008	4.333±0.918	<0.0001	1.997±0.508	0.0015
	ALS	1.062±0.617		0.7099±0.564		0.2512±0.237	
Lymphatic tonic index	NC	6.679±1.000	0.0462	6.441±0.981	0.0543	7.025±1.459	0.0128
	ALS	3.786±1.606		3.246±1.291		3.16±1.327	

*All means were compared using Mann–Whitney test. ALS, amyotrophic lateral sclerosis; NC, non-transgene carrier; P, pressure.

### Association between inflammation and lymphangiogenesis gene marker expression in skeletal muscle and SPC from SOD1-G93A mice

**Fig. 3.**
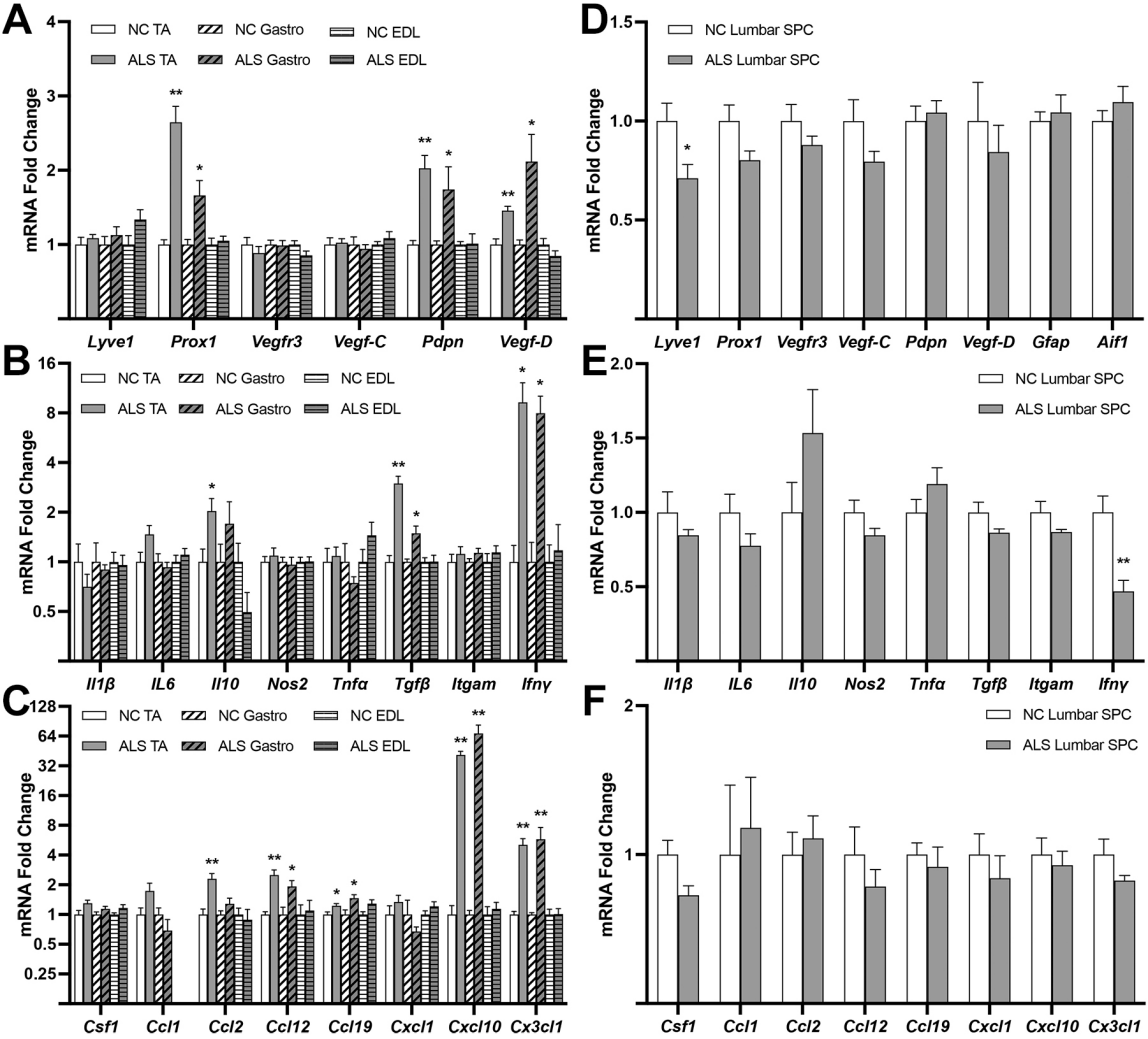
**Lymphatic gene marker mRNA induction is linked to inflammation in skeletal muscle of presymptomatic SOD1-G93A mice.** (A-F) Quantitative real-time PCR of mRNA for lymphatic and inflammation gene markers for hindlimb skeletal muscles [extensor digitorum longus (EDL), gastrocnemius (Gastro), tibialis anterior (TA)] (A-C) and lumbar spinal cord (D-F) from p50 NC and ALS SOD1-G93A mice. *n*=6 males/genotype. Values are mean±s.e.m. *P<0.05, **P<0.01. Unpaired two-tailed *t*-test was used to compare means for all markers except for *Ccl1* (lumbar SPC); *Vegfr3*, *Cxcl1*, *Clcx10* (TA); *Il10*, *Tnfa*, *Ifng*, *Ccl12*, *Cxcl1*, *Cxcl10*, *Cx3cl1* (Gastro); *Pdpn*, *Ccl2*, *Ccl12* (EDL), for which means were compared using Mann–Whitney test. *Ccl1* mRNA was undetectable in the EDL samples.

**Fig. 4.**
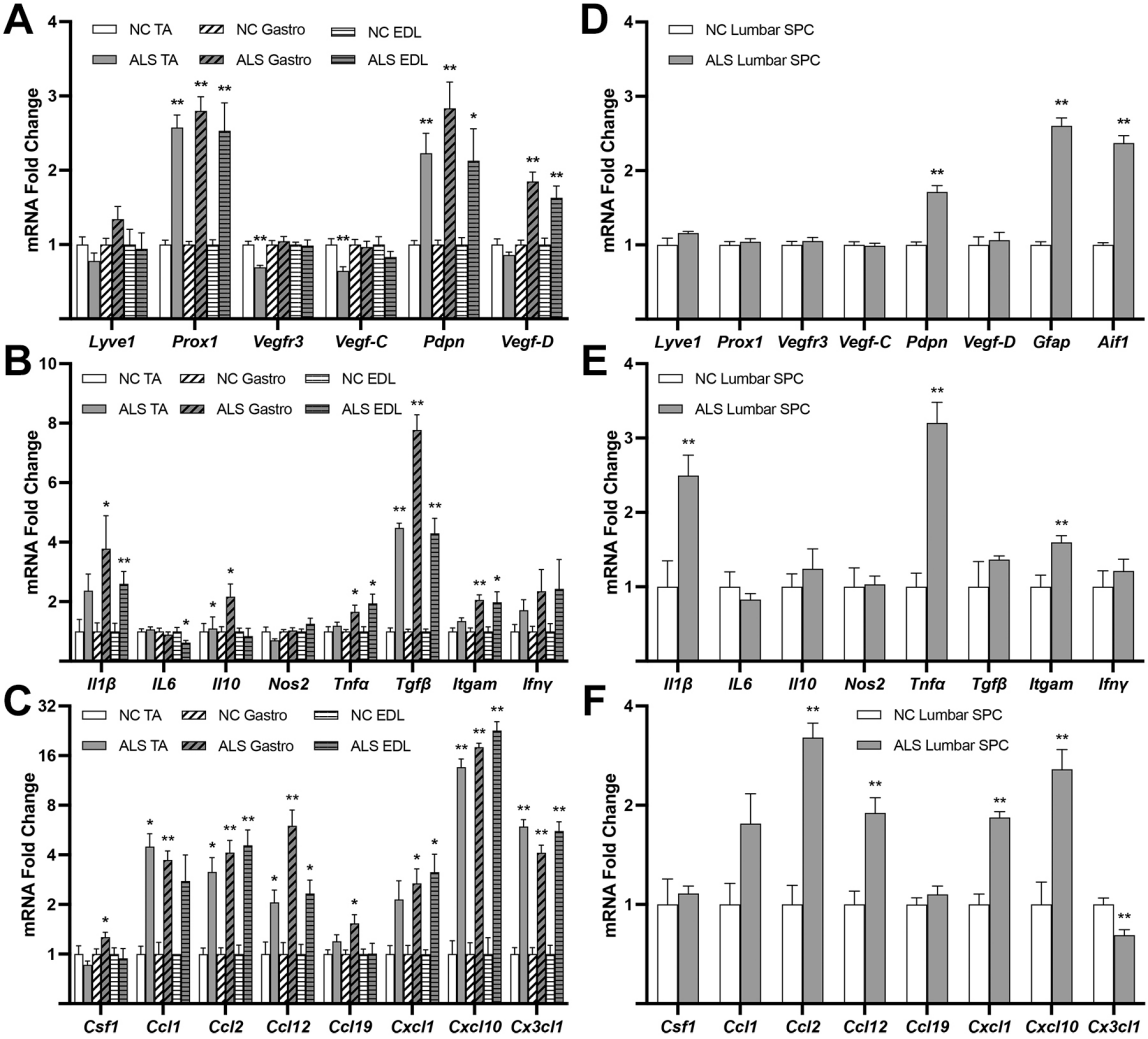
**Lymphatic gene marker mRNA induction is linked to inflammation in skeletal muscle and spinal cord of postsymptomatic SOD1-G93A mice.** (A-F) Quantitative real-time PCR of mRNA for lymphatic and inflammation gene markers for hindlimb skeletal muscles (EDL, Gastro, TA) (A-C) and lumbar spinal cord (D-F) from p120 NC and ALS SOD1-G93A mice. *n*=6 males/genotype. Values are mean±s.e.m. *P<0.05, **P<0.01. Unpaired two-tailed *t*-test was used to compare means for all markers except for *Il6*, *Nos2*, *Tgfb*, *Csf1* (lumbar SPC); *Ccl2*, *Cxcl1* (TA); *Prox1*, *Il1b*, *Ifng*, *Cxcl1* (Gastro); *Ccl2*, *Cxcl1*, *Cx3cl1* (EDL), for which means were compared using Mann–Whitney test.

Systemic inflammation is a hallmark feature in ALS animal models and the human disease (Appel et al., 2021). Moreover, in many other models of systemic inflammatory diseases, pathological lymphangiogenesis is associated with inflammation and lymphatic dysfunction (Fogt et al., 2004; Detmar et al., 1994; Wauke et al., 2002; Pedica et al., 2008; Tabibiazar et al., 2006). Hence, we used quantitative real-time PCR to first determine changes in mRNA expression levels of lymphatic and inflammatory gene markers in affected skeletal muscles and SPC in SOD1-G93A mice relative to those in control mice at both presymptomatic and postsymptomatic stages (p50 and p120, respectively). For these experiments, the SPC tissue was carefully dissected to maximize retention of the meninges. Fig. 3A shows induction of lymphatic markers *Prox1* and *Pdpn* in two of the three p50 ALS hindlimb muscles examined: gastrocnemius (Gastro) and tibialis anterior (TA) (ALS vs NC; *Prox1* mRNA fold changes: Gastro, 1.661±0.201, *P*=0.011; TA, 2.645±0.215, *P*=2.52×10^−5^; *Pdpn* mRNA fold changes: Gastro, 1.74±0.306, *P*=0.038; TA, 2.028±0.171, *P*=0.001). Another important marker, the lymphangiogenic factor *Vegfd* (Rissanen et al., 2003), was increased in p50 Gastro and TA (ALS vs NC; Gastro, 2.117±0.366, *P*=0.013; TA, 1.457±0.059, *P*=0.001). mRNA levels of other lymphatic markers – *Lyve1*, *Vefgr3* and *Vegfc* – were unchanged in the ALS muscles relative to NC control muscles, and the p50 extensor digitorum longus (EDL) muscle failed to show any mRNA changes in lymphatic markers relative to control EDL (Fig. 3A). Transcripts for cytokines (Fig. 3B) and chemokines (Fig. 3C), used here as inflammation markers, were unaltered in the p50 ALS EDL, whereas those for selected cytokines [*Il10*, *Tgfb* (also known as *Tgfb1*), *Ifng*] and chemokines (*Ccl12*, *Ccl19*, *Cxcl10*, *Cx3cl1*) were significantly elevated in p50 TA and Gastro. Although *Lyve1* was slightly reduced (0.711±0.069, *P*=0.029), none of the other lymphatic markers, and all but one (*Ifng*) of the inflammation markers, including astrogliosis marker *Gfap* and microgliosis marker *Aif1*, coding for Iba1, were changed in p50 lumbar SPC relative to NC control lumbar SPC (Fig. 3D-F). Fig. 4A shows induction of *Prox1* and *Pdpn* in p120 ALS EDL, Gastro and TA (ALS vs NC; *Prox1* mRNA fold changes: EDL, 2.530±0.376, *P*=0.002; Gastro, 2.800±0.189, *P*=3.13×10^−6^; TA, 2.574±0.169, *P*=5.2×10^−6^; *Pdpn* mRNA fold changes: EDL, 2.130±0.429, *P*=0.028; Gastro, 2.833±0.355, *P*=4.68×10^−4^; TA, 2.230±0.266, *P*=0.001). *Vegfd* was increased in p120 ALS EDL and Gastro (ALS vs NC; EDL, 1.629±0.158, *P*=0.006; Gastro, 1.849±0.126, *P*= 1.17×10^−4^). Inflammation was confirmed in all three p120 ALS muscles by increases in various key cytokines (Fig. 4B) and chemokines (Fig. 4C). *Lyve1*, *Vefgr3 and Vegfc* remained unchanged in p120 ALS muscle except for a slight reduction in the TA for *Vefgr3* and *Vegfc* (Fig. 4A). In p120 ALS lumbar SPC, *Pdpn* mRNA was significantly increased relative to that in p120 control lumbar SPC (1.713±0.086, *P*=1.95×10^−5^), together with mRNA levels of markers of astrogliosis (*Gfap*, 2.602±0.108, *P*=7.4×10^−8^) and microgliosis (*Aif1*, 2.371±0.101, *P*=1.4×10^−7^) (Fig. 4D). None of the other lymphatic markers were changed in p120 ALS lumbar SPC compared to that in p120 control lumbar SPC. Increases in mRNA levels for selected cytokines (Fig. 4E) and chemokines (Fig. 4F) in p120 ALS SPC confirmed its expected proinflammatory status. Thus, lymphatic marker increases in the ALS mice occurred in tissues in which inflammation markers also increased (p50 Gastro and TA; p120 EDL, Gastro, TA and lumbar SPC) but not in those in which the latter markers were unaltered (p50 EDL and lumbar SPC).

**Fig. 5.**
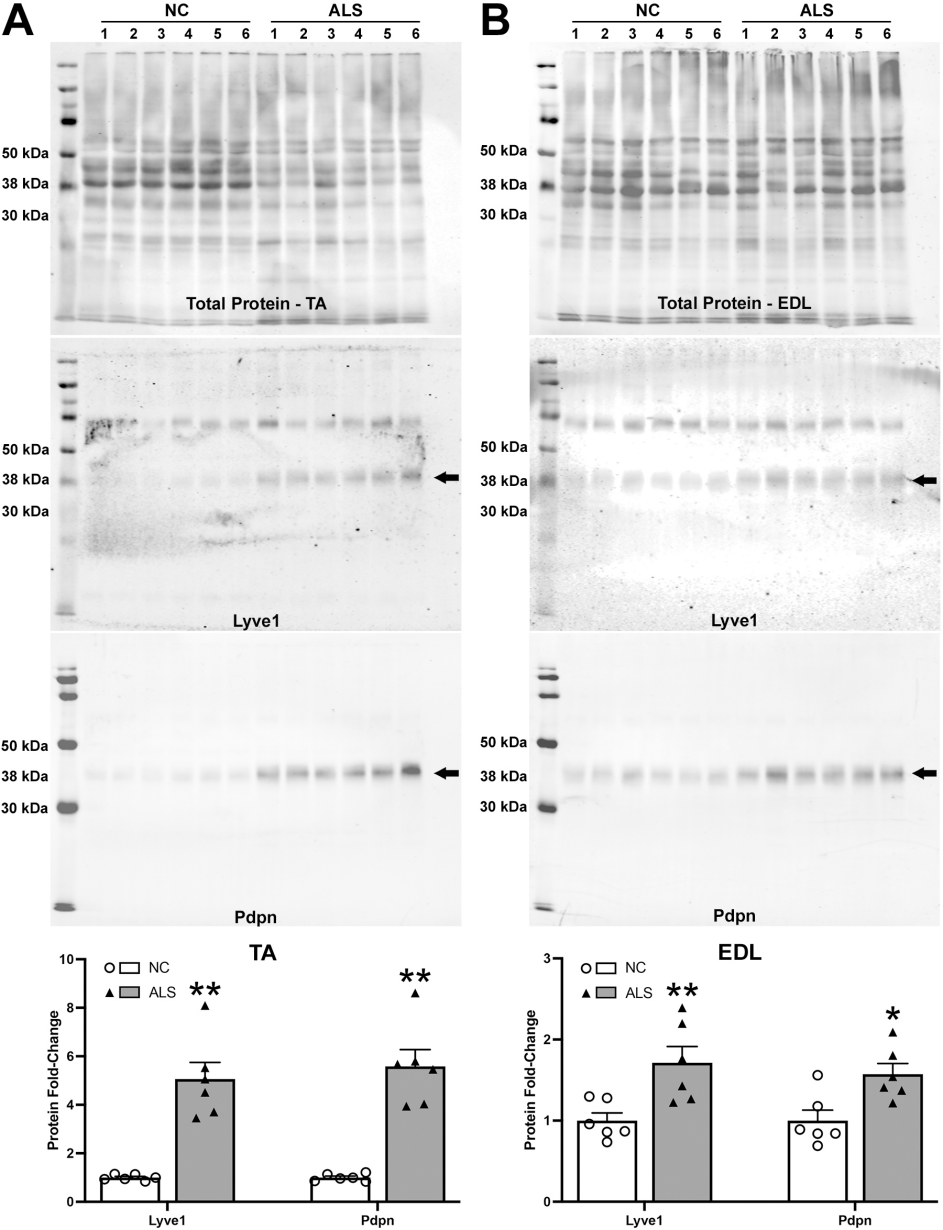
**Western blots for p120 TA and EDL probed for Lyve1 and Pdpn.** (A) p120 TA. (B) p120 EDL. Top panels show total loaded protein, 30 μg/lane, as stained with Revert 700 total protein stain (LI-COR). Arrows in middle panels point to expected bands for each protein, as predicted by their molecular mass in kDa. Other bands in Lyve1 blots are non-specific. Histograms in bottom panels show quantification of signal normalized to total protein/lane and expressed relative to control. The data points in the histograms represent individual muscles, each from different mice (*n*=6/genotype, males). Data presented as mean±s.e.m. *P<0.05, **P<0.01. Means for TA data were compared with Mann–Whitney test; means for EDL data were compared with unpaired two-tailed *t*-test.

We next used western blotting to analyze the expression of lymphatic markers in p120 skeletal muscle at the protein level. We probed for Lyve1 and Pdpn in both p120 EDL and TA. Fig. 5 shows that Pdpn and Lyve1 were significantly elevated in both ALS muscles (ALS vs NC; Pdpn fold changes: EDL, 1.573±0.131, *P*=0.011; TA, 5.587±0.691, *P*=6×10^−5^; Lyve1 fold changes: EDL, 1.713±0.201, *P*=0.010; TA, 5.060±0.686, *P*=1.5×10^−4^). Although the elevation in Pdpn protein in ALS muscles was in good correlation with the increase at the mRNA level observed above (Fig. 4), Lyve1 protein increased in ALS EDL and TA (Fig. 5), whereas *Lyve1* mRNA failed to change relative to that in control in any of the three ALS muscles examined (Fig. 4). This suggests that transcriptional mechanisms control *Pdpn* expression, whereas post-transcriptional mechanisms regulate Lyve1 protein levels in ALS muscle. Thus, together, these results suggest a link between inflammation and lymphatic marker upregulation in the SOD1-G93A ALS mice, similar to what has been found in other inflammatory diseases.

### Lymphatic capillary network coverage in lumbar SPC and EDL muscle appears largely unaltered in SOD1-G93A mice

**Fig. 6.**
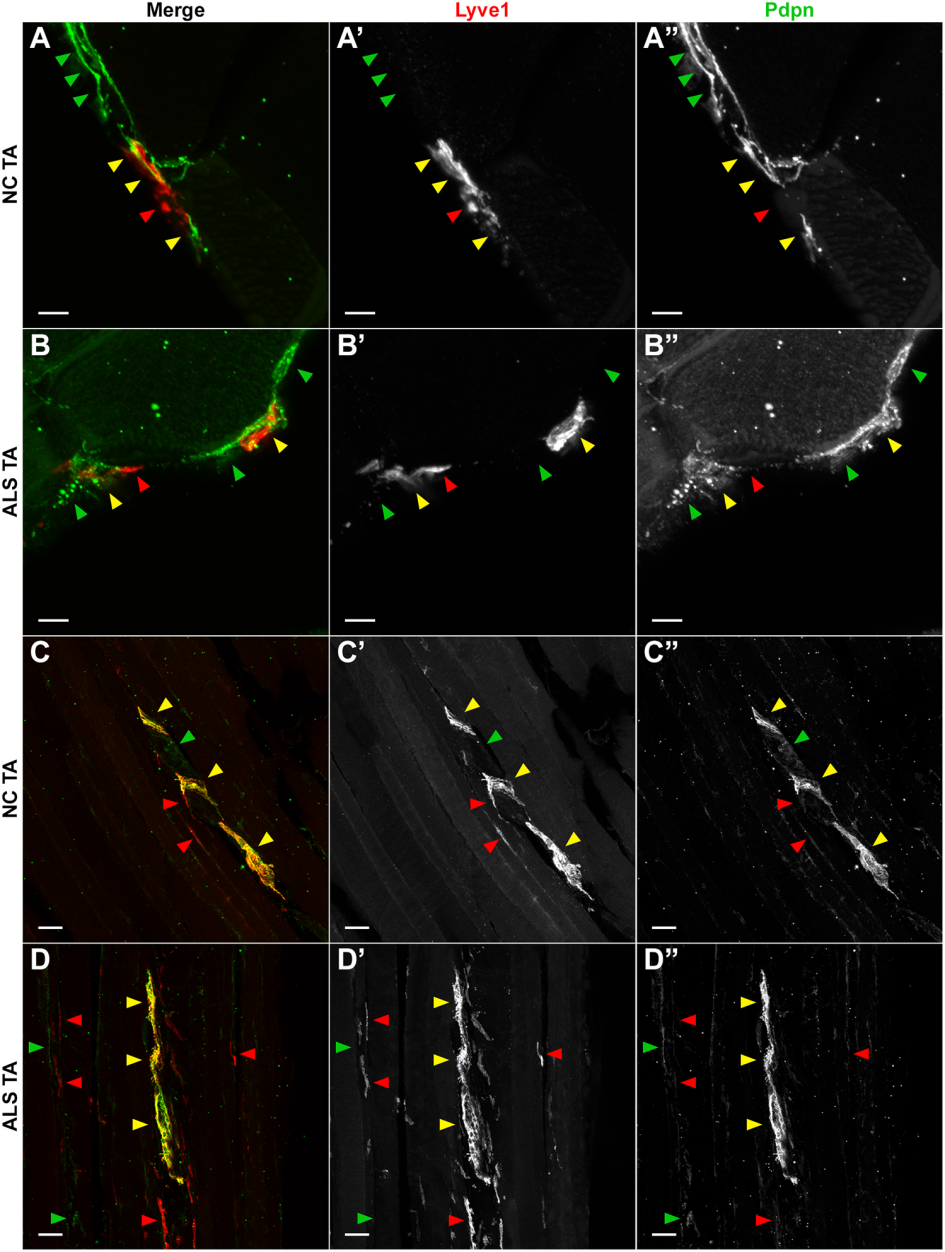
**Detection of lymphatic vessels in skeletal muscle with antibodies against Lyve1 and Pdpn.** (A) Maximal-intensity projection (MIP) of a representative cross-section of a p120 control NC TA double-labeled for Lyve1 and Pdpn, imaged in a confocal microscope and pseudocolored red (Lyve1) and green (Pdpn). Yellow arrowheads point to regions of staining overlap; green and red arrowheads point to Pdpn and Lyve1 staining, respectively, where there is no staining overlap. (A′) Grayscale Lyve1 staining. (A″) Grayscale Pdpn staining. (B) MIP of a representative cross-section of a p120 ALS TA double-labeled for Lyve1 and Pdpn, imaged and pseudocolored as in A, with two areas of partial colocalization of Lyve1 and Pdpn staining. (B′) Grayscale Lyve1 staining. (B″) Grayscale Pdpn staining. Owing to green autofluorescence of muscle fibers, here it can be better appreciated that the Lyve1/Pdpn staining is detected outside muscle fibers. (C) MIP of a representative longitudinal section of a p120 NC TA that was double-labeled for Lyve1 and Pdpn, imaged and pseudocolored as in A. Vessel-like structures, running parallel to the muscle fibers can be seen co-labeling for Lyve1 and Pdpn (yellow arrowheads) or singly labeled for each marker. Red arrowheads, Lyve1; green arrowheads, Pdpn. (C′) Grayscale Lyve1 staining. (C″) Grayscale Pdpn staining. (D) MIP of a representative longitudinal section of an p120 ALS TA that was double-labeled for Lyve1 and Pdpn, imaged and pseudocolored as in A. (D′) Grayscale Lyve1 staining. (D″) Grayscale Pdpn staining. Scale bars: 5 μm (A-B″); 50 μm (C-D″).

We first tried to confirm the specificity of our antibodies for lymphatic vessels by co-staining transverse and longitudinal sections of control and ALS TA muscles for Lyve1 and Pdpn. We expected colocalization of the two stains on vessel-like structures that would run mostly parallel to muscle fibers. Fig. 6 (yellow arrowheads) shows that this was indeed the case. However, we also found vessel-like structures that were only either Lyve1^+^ or Pdpn^+^ (Fig. 6, red and green arrowheads, respectively). Pdpn^+^ collecting lymphatics in dermal preparations have been reported as having low to absent Lyve1 staining (Ulvmar and Makinen, 2016; Mäkinen et al., 2005), while in tongue muscle (Noda et al., 2010), in addition to colocalizing with Lyve1 at bona fide lymphatics, Pdpn staining was observed as well in intramuscular nerve epineurium, which has also been shown to harbor lymphatic vessels identified only by positive Pdpn staining (Volpi et al., 2006). Lyve1^+^/Pdpn^−^ lymphatic vessels were found in normal lung parenchyma (Renyi-Vamos et al., 2005). Thus, from these experiments, we concluded that our antibodies against Lyve1 and Pdpn were indeed staining lymphatic vessels, but that, at least in skeletal muscle, there also seems be a heterogeneity of lymphatics regarding their labeling with classical lymphatic markers.

**Fig. 7.**
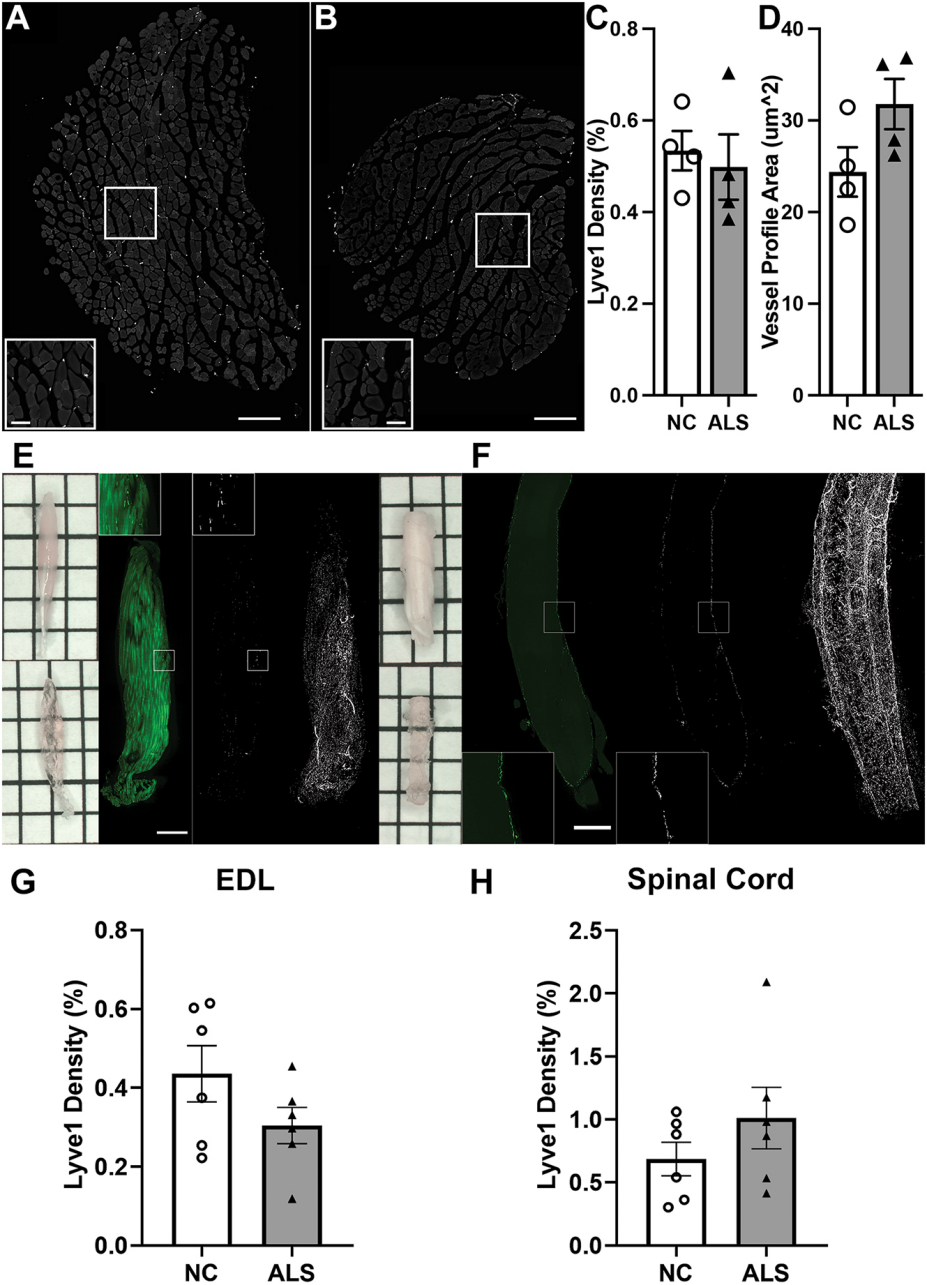
**Lymphatic capillary coverage in lumbar spinal cord and EDL muscle appears largely unaltered in SOD1-G93A mice.** (A) Representative cross-section of p120 NC EDL muscle stained for Lyve1. Scale bar: 200 μm. Inset: higher-magnification view. Scale bar: 50 μm. (B) Representative cross-section of p120 ALS EDL muscle stained for Lyve1. Scale bar: 200 μm. Inset: higher-magnification view. Scale bar: 50 μm. (C) Lyve1 density (Lyve1 staining area/area of cross-section×100%) (*n*=4 males/genotype; *P*=0.6875). (D) Vessel profile area (μm^2^). (E) Muscle tissue clearing before (top left) and after (bottom left) iDISCO protocol. Representative lightsheet slice of p126 NC EDL tissue labeled for Lyve1 (left, raw Lyve1 staining; middle, thresholded Lyve1 signal; right, MIP of thresholded Lyve1 image). Scale bar: 1 cm. (F) Representative lightsheet slice of p126 NC lumbar spinal cord tissue labeled for Lyve1 (left, raw Lyve1 staining; middle, thresholded Lyve1 signal; right, MIP of thresholded Lyve1 image). Scale bar: 1 cm. (G) Lyve1 density in thresholded lightsheet images in EDL tissue (*n*=6 males/genotype; *P*=0.1597). (H) Lyve1 density in thresholded lightsheet images in spinal cord tissue (*n*=6 males/genotype; *P*=0.2784). All means in C, D, G and H compared by unpaired two-tailed *t*-test.

The increases, particularly in *Vegfd* mRNA, and Lyve1 and Pdpn protein levels in p120 EDL (Fig. 4A and Fig. 5B, respectively) and of *Pdpn* mRNA in p120 lumbar SPC (Fig. 4C), in the context of elevated inflammation (Fig. 4), suggested an expansion in lymphatic capillary coverage, i.e. inflammatory lymphangiogenesis, in these two tissues in ALS mice. To examine this directly, we stained transverse sections of the EDL muscle for Lyve1 and then quantified the staining as described in the Materials and Methods. As expected, Lyve1^+^ profiles of lymphatic vessels were found between muscle fibers in both NC and ALS EDL (Fig. 7A,B). Surprisingly, Lyve1 staining density per ALS EDL cross-section was not different from that in NC control EDL (Fig. 7C), despite the muscle having higher Lyve1 protein levels overall (Fig. 5B) (NC, 0.534±0.043%; ALS, 0.499±0.071%; *P*=0.6875). On the other hand, quantification of the individual vessel area showed that, on average, profiles of ALS capillaries tended to be bigger than those of NC capillaries in the EDL (Fig. 7D) (NC, 24.384±2.697 μm^2^; ALS, 31.780±2.742 μm^2^; *P*=0.100), which suggested circumferential growth (Alitalo, 2011) as a mechanism to increase the size of lymphatic capillaries in ALS muscle. To further examine lymphatic vessel coverage, we turned to iDISCO, a whole-mount staining approach using tissue-clearing techniques combined with lightsheet fluorescence microscopy (Jalufka et al., 2022), which allowed us to visualize and quantify Lyve1 staining in the tissue in its entirety rather than by selected sections as above. In the EDL, we found a modest, albeit non-significant, trend towards a reduction in Lyve1 staining density (Fig. 7E,G) (NC, 0.435±0.072%; ALS, 0.304±0.046%; *P*=0.159). When iDISCO tissue clearing and 3D lightsheet imaging were applied to a preparation of the lumbar SPC in which meninges were preserved (see Materials and Methods), we found that Lyve1 density, although highly variable, trended higher in ALS tissue than in control tissue (Fig. 7F,H) (NC, 0.686±0.133%; ALS, 1.011±0.245%; *P*=0.278). Movies 1 and 2 show representative 3D videos of the above observations with iDISCO, while Fig. S2 shows that Lyve1 density staining quantification in p126 ALS female tissue exhibited the same trends as in male tissue (Fig. 7F,H). Thus, lymphatic capillary density appeared modestly altered overall, slightly decreased in p120-p126 ALS EDL muscle, but slightly increased in p126 lumbar ALS SPC, relative to that in control EDL muscle and SPC, respectively.

## DISCUSSION

Here, we show, for the first time, that presymptomatic and postsymptomatic SOD1-G93A ALS mice exhibit lymph transport dysfunction and an association between inflammation and lymphatic marker upregulation in key affected tissues in the disease, skeletal muscle and SPC. In the postsymptomatic stage, SOD1-G93A ALS mice also exhibited diminished intrinsic collecting lymphatic vessel contraction. However, we were unable to detect major alterations (i.e. expansion or regression) in lymphatic capillary coverage in lumbar SPC and in one affected muscle we studied in detail, the EDL.

There was a close correlation between hindlimb lymphatic transport dysfunction (Fig. 1) and upregulation of lymphatic and inflammatory markers in the hindlimb skeletal muscle groups analyzed (Figs 3, 4 and 5), which could be observed even in the clinically presymptomatic stage (p50) (Fig. 3). These results add to a known series of cell and molecular abnormalities found in SOD1-G93A mice at the clinically presymptomatic stage (e.g. Vinsant et al., 2013; Chiu et al., 2009), and raise the possibility that defective lymph transport and/or upregulation of lymphatic gene markers in skeletal muscle biopsies could be further investigated in the future as early biomarkers of disease.

Our findings of much less efficient hindlimb lymph transport in ALS mice (Fig. 1) are consistent with the notion that skeletal muscle contraction may be one of the driving forces for lymph propulsion (Alitalo, 2011), as muscle denervation, even in some specific muscle groups in the clinically presymptomatic period (Cantor et al., 2018; Vinsant et al., 2013), is a cardinal feature of the disease in these animals and in human patients (Fischer et al., 2004). This raises the interesting, yet largely unexplored, possibility that many neuromuscular disorders with neurogenic or myogenic muscle dysfunction exhibit at least some defective lymph transport phenotype. However, the decrease in hindlimb lymph transport that we found in symptomatic ALS mice better correlates with the severely diminished intrinsic contractile activity we measured in isolated collecting flank lymphatic vessels from ALS mice (Fig. 2). In other disease models, this specific phenotype has also been associated with elevation of inflammation (e.g. Chakraborty et al., 2015; Chen et al., 2017; Lee et al., 2020), which could well be the case in symptomatic SOD1-G93A mice (e.g. Fig. 4). In this context, and supporting an association between lymphatic dysfunction and inflammation, at least in some forms of ALS, progressive lymphadenopathy linked to neuroinflammation and altered macrophage and microglia immune responses, despite normal muscle electrophysiology, limb strength and absence of neurodegeneration, was reported in *C9orf72^−/−^* mice, a model for the most common form of familial ALS (O'Rourke et al., 2016).

We found that intrinsic phasic and tonic contractile activity was diminished in ALS flank lymphatic vessels relative to that in control flank lymphatic vessels (Fig. 2 and Table 1). Reactive oxygen species (ROS) decreased ejection fraction, contraction frequency and lymph pump flow in rat mesenteric lymphatic vessel preparations (Zawieja et al., 1991). As elevated ROS levels have been reported in skeletal muscles of SOD1-G93A mice, even before symptom onset (Xiao et al., 2018), it is possible that increased levels of ROS in ALS mice directly influence the contractile activity of lymphatic vessels. Our data also showed an increase in *Itgam* in postsymptomatic muscle and SPC (Fig. 4), suggesting an increase in CD11b^+^ immune cells, which would have decreased lymphatic contractile activity via an inducible nitric oxide synthase mechanism (Liao et al., 2011). In addition, these effects are reminiscent of our previous report in mesenteric lymphatic vessels in a rat model of metabolic syndrome (Lee et al., 2020). We showed that lymphatic muscle cells express SERCA2 (also known as ATP2A2) isoforms, SERCA2a and SERCA2b, but only the levels of SERCA2a were reduced in a high-fructose diet rat model of metabolic syndrome. Selective pharmacological activation of SERCA2 partially rescued the contractile activity of lymphatic vessels in the metabolic syndrome rats (Lee et al., 2020). In view of these results, and given the recent findings that transgenic overexpression of SERCA1 (also known as ATP2A1) in skeletal muscle mitigates muscle atrophy and enhances motor function in SOD1-G93A mice (Mazala et al., 2024), in future mechanistic studies it will be important to examine whether SERCA2 isoform expression is also selectively reduced in murine SOD1-G93A lymphatic vessels, and whether its pharmacological activation has any effects on lymphatic function and, more importantly, on disease progression.

Although several previous studies have analyzed lymphatic network coverage in wild-type rodent skeletal muscle (Mazzoni et al., 1987; Rissanen et al., 2003; Kawashima et al., 2021; Ji, 2018; Taketa et al., 2024; Wardrop and Dominov, 2011) and, more recently, SPC (Jacob et al., 2019; Gonuguntla and Herz, 2023), ours is the first we are aware of that has examined it in the context of an ALS animal model. It is noteworthy that we found no changes in the expression of *Vegfc* and *Vegfr3* in ALS muscle and lumbar SPC, except for a small decrease in the p120 ALS TA (Fig. 4A), even though *Il1b* and *Tnfa* (also known as *Tnf*), proinflammatory cytokines that upregulate *Vegfc* expression (Ristimäki et al., 1998), were upregulated as well postsymptomatically (Fig. 4B,E). Because we only examined *Il1b* and *Tnfa* at the mRNA level, it remains undetermined whether there was a corresponding increase at the protein level, sufficient to drive *Vegfc* expression higher. *Vegfc* is essential for the formation of peripheral (Karkkainen et al., 2004) and meningeal lymphatics during development (Antila et al., 2017), and the fact that its expression is unaltered in the ALS mice may account for the minimal changes in lymphatic capillary coverage we report in postsymptomatic SPC and muscle. However, increased release of VEGF-C, which we did not measure here, could have resulted from the rise in *Ccl2* mRNA (Fig. 4) (Chen et al., 2018). Moreover, VEGF-D was the strongest ectopic lymphangiogenic factor in adult skeletal muscle (Rissanen et al., 2003), and we found *Vegfd* mRNA expression to be elevated in presymptomatic and postsymptomatic ALS muscle (Figs 3 and 4). We found significant increases in *Tgfb*, and a trend to higher levels of *Ifng*, mRNA in all three p120 ALS muscles studied (Fig. 4). IFNγ and TGF-β have been implicated in lymphatic vessel regression in other experimental systems (Masood et al., 2022; Shi et al., 2020; Kataru et al., 2011). The potential anti-lymphangiogenic activity of these factors might counterbalance the lymphangiogenic activity of VEGF-D in ALS EDL muscle and contribute to the slight reduction we observed in lymphatic capillary density in this muscle (Fig. 7), despite the net increase in lymphatic markers at the mRNA and protein levels (Figs 4 and 5). We detected an upward trend in circumferential growth in lymphatic capillaries in ALS EDL (Fig. 7D) that, in part, might account for the increase in lymphatic vessel proteins (Fig. 5). On the other hand, the slight trend towards an increase in lymphatic capillary coverage we found in lumbar ALS SPC (Fig. 7) was consistent with the statistically significant rise in p120 *Pdpn* mRNA in that tissue (Fig. 4). Evidence from other experimental systems implicate additional molecules that could modulate lymphangiogenesis in SOD1-G93A mice, which we have not studied here [e.g. galectin-8 (Chen et al., 2016); syndecan-4 (Johns et al., 2016)]. Although the known severity of its ALS phenotype, the increase in expression of lymphangiogenic factors at the mRNA and protein levels (Figs 4 and 5), and its ideal size for iDISCO made us select the EDL for analysis of lymphatic capillary density here, we cannot rule out that other muscles in the SOD1-G93A mice show a different response as far as lymphatic network coverage is concerned. It will be interesting to study lymphatic capillary density and lymphatic marker expression in muscles like the slow-twitch soleus, which in SOD1-G93A mice are affected by the disease at much later stages than the fast-twitch EDL or TA (e.g. Harrison and Rafuse, 2020). An additional limitation in our lymphatic vessel coverage studies comes from using only Lyve1 staining as a measure of lymphatic density changes. Although staining for Lyve1 is widely used in the field to characterize lymphatic capillary density, and we showed Lyve1/Pdpn colocalization in vessel-like structures in skeletal muscle that validates our Lyve1 antibodies (Fig. 6), these experiments also revealed a possible heterogeneity in lymphatic vessels (for example, Pdpn^+^/Lyve1^−^ collectors vs Pdpn^+^/Lyve1^+^ capillaries) that we have failed to account for in the present work. Although much of the Lyve1 staining we observed in TA (Fig. 6) and in iDISCO EDL and lumbar SPC preparations (Fig. 7; Movies 1 and 2) appeared on vessel-like structures and not on single cells, we cannot rule out that some of the Lyve1 staining we detected was on single cells like macrophages, which have been reported to also stain for Lyve1 in tongue muscle (Noda et al., 2010). Lastly, it is worth mentioning in this context that attempts to use Pdpn staining within the iDISCO protocol have failed so far in our hands for unknown technical reasons likely related to the unsuitability of the anti-Pdpn antibody for this approach.

Adaptive changes in lymphatic function and lymphangiogenesis are associated with peripheral acute and chronic inflammation (Alitalo, 2011). However, it is unclear whether they are part of a protective response or of the pathology itself (Vranova and Halin, 2014). For example, in inflammatory bowel disease, the accompanying lymphangiogenesis appears protective because its enhancement improves disease course by facilitating the resolution of inflammation through promoting immune cell mobilization (D'Alessio et al., 2014), whereas its blockage makes disease worse (Jurisic et al., 2013). By contrast, rejection of heterologous transplantation, which is accompanied by extensive lymphatic network growth, is mitigated by blocking lymphangiogenesis (e.g. Dietrich et al., 2010). The recent ‘rediscovery’ of meningeal lymphatics in the CNS (Louveau et al., 2015; Aspelund et al., 2015) has stimulated investigations into the possible role of central lymphatics in neurodegenerative diseases that feature prominent neuroinflammation such as Alzheimer's disease (AD), with somewhat conflicting results. For example, one study found that meningeal lymphatic morphology and function was not altered relative to control in the APeD9 and 5xFAD mouse models of AD, and although manipulating VEGF-C levels and signaling in these mice led to expected changes in meningeal lymphatics coverage and drainage function to cervical lymphatics nodes, it failed to have an impact on amyloid plaque accumulation or behavioral AD-like phenotypes (Antila et al., 2024). On the other hand, another study on 5xFAD mice found that ablation of meningeal lymphatics exacerbated amyloid-β deposition and behavioral phenotypes, while augmenting meningeal lymphangiogenesis led to improved clearance of amyloid-β by immunotherapy (Da Mesquita et al., 2021). Now that we have shown, for the first time, that SOD1-G93A mice exhibit lymphatic dysfunction and a seemingly limited lymphangiogenesis in SPC and muscle, it will be important to follow up with experiments that test the effects of increasing or blocking lymphangiogenesis in skeletal muscle and SPC on ALS disease course. It will also be important to examine whether the lymphatic phenotypes we have unraveled here extend to other animal models of ALS and, most importantly, to human patients.

## MATERIALS AND METHODS

### Mice and motor phenotyping

SOD1-G93A transgenic mice of the C57BL/6J background were obtained from The Jackson Laboratory (JAX 004435). A colony of these animals was established locally by crossing hemizygous transgenic males with C57BL/6J females. Mice were housed on a 12 h light/dark cycle with *ad libitum* access to food and water. Their genotype was established following protocols recommended by The Jackson Laboratory. Unless otherwise indicated, only male mice from the local colony or directly acquired from The Jackson Laboratory, and their C57BL/6J age- and sex-matched NC controls, were used for experiments. Starting at p35, body weight, forelimb grip strength, hindlimb splay and tremor were assessed weekly in a subset of the animals from the local colony. Forelimb grip strength was measured as previously described (Seaberg et al., 2015). Hindlimb tremor and splay were scored using the scales defined in Mead et al. (2011). These data were averaged per genotype (Fig. S1). End stage was defined as failure to righting after 15 s or loss of 20% body weight relative to week 12 of life (Sengupta-Ghosh et al., 2018). Care and treatment of all animals followed the National Institutes of Health Guide for the Care and Use of Laboratory Animals, and were approved by the Institutional Animal Care and Use Committee of Texas A&M University under animal use protocols 2021-0334-D and 2024-0278-D.

### Microlymphangiography

Fluorescence microlymphangiography is a non-invasive method used to visualize lymph transport in initial capillaries and deeper collecting lymphatic vessels in the right hind limb of mice. Mice were anesthetized with isoflurane at an oxygen flow rate of 0.8 l/min. After shaving the hair around the hindlimb region, the footpad of the mice was injected with 10 μl of 2 mg/ml fluorescein-conjugated dextran (FITC-Dextran) intradermally with a 30-gauge needle. Care was taken to minimize potential swelling or tissue damage and facilitate physiologic lymphatic uptake. The FITC-Dextran travels through the interstitial space in which the lymphatic capillaries take it up. Lymph transport was monitored on a fluorescence stereomicroscope for a total of 30-60 min, with images taken every 5 min (Rutkowski et al., 2006). The time taken for transport was observed, and the amount of lymph transported was inferred by measuring the fluorescence intensity of a standardized area of interest at every time point using ImageJ software. The intensity was standardized to the area of interest using the formula mean [raw integrated density/area of region of interest (ROI)], keeping the ROI constant within each animal. This was then normalized to the NC controls.

### Lymphatic vessel isolation, cannulation and functional analysis *ex vivo*

Mouse flank collecting lymphatic vessel isolation and cannulation was performed to assess their contractile function at varying levels of applied transmural pressure, as described in our previous studies (Lee et al., 2020). Briefly, mice were euthanized by exsanguination under isoflurane, and flank lymphatic vessels were carefully isolated, ensuring thorough removal of surrounding adipose and connective tissues by microdissection. The isolated lymphatic vessel was then transferred to an observation chamber with Krebs-bovine serum albumin (BSA) buffer, where it was cannulated and pressurized at 1.0 cmH_2_O pressure using micropipettes in a CH-1 chamber for 30 min at 37°C. After equilibration, the vessel was video recorded using a firewire camera for 5 min at 1.0, 2.0 and 4.0 cmH_2_O of pressure. Lastly, the buffer was replaced with a calcium-free Krebs buffer containing 3 mM EGTA for 30 min to obtain the passive, maximal diameter (*D*_max_) at each pressure. Throughout the experiment, the lymphatic outer diameters were recorded as a function of time via the videos using Vessel Track software (Davis, 2005). The outer diastolic diameter (ODD), outer systolic diameter (OSD) and contraction frequency were obtained directly from Vessel Track LabVIEW software (provided by Dr Michael J. Davis, University of Missouri, Columbia, MO, USA). The associated contractile parameters that were calculated are comparable to those of the cardiac pump functions: ejection fraction (EF)=(ODD^2^−OSD^2^/ODD^2^); fractional pump flow=EF×contractile frequency; amplitude=(ODD−OSD/*D*_max_)×100; and lymphatic tonic index=(*D*_max_−ODD/*D*_max_)×100.

### Quantitative real-time PCR

Total RNA extraction, reverse transcription and real-time PCR were performed essentially as previously described (Wang et al., 2016). A DNase treatment step was added before reverse transcription according to the manufacturer's instructions (TURBO DNA-free Kit, Invitrogen, AM1907). cDNA samples were set up in duplicate using TaqMan Master Mix (Thermo Fisher Scientific) manufacturer protocols and run for 50 cycles on an Applied Biosystem ViiA 7 Real-time PCR system (ABI/Life Technologies/Thermo Fisher Scientific). All TaqMan primer/probe sets were from Thermo Fisher Scientific/Life Technologies: *18s* rRNA (4333760F), *Lyve1* (Mm00475056_m1), *Prox1* (Mm00435969_m1), *Vegfr3* (Mm01292604_m1), *Vegfc* (Mm00437310_m1), *Pdpn* (Mm01348912_g1), *Vegfd* (Mm01131929_m1), *Gfap* (Mm01253033_m1), *Aif1* (Mm00479862_g1), *Il1b* (Mm00434228_m1), *Il6* (Mm00446190_m1), *Il10* (Mm01288386_m1), *Nos2* (Mm00440502_m1), *Tnfa* (Mm00443258_m1), *Tgfb* (Mm01178820_m1), *Itgam* (Mm00434455_m1), *Ifng* (Mm01168134_m1), *Csf1* (Mm00432686_m1), *Ccl1* (Mm00441236_m1), *Ccl2* (Mm00441242_m1), *Ccl12* (Mm01617100_m1), *Ccl19* (Mm00839966_g1), *Cxcl1* (Mm04207460_m1), *Cxcl10* (Mm00445235_m1), *Cx3cl1* (Mm00436454_m1). *18s* rRNA cycle threshold (Ct) values were used to equalize total RNA levels between samples, and expression level changes were determined using the ddCT method (Livak and Schmittgen, 2001), normalizing the results relative to the NC controls (set to 1).

### Western blotting

Muscles were extracted in RIPA buffer (50 mM Tris-HCl pH8.0, 150 mM NaCl, 0.1% SDS, 0.5% sodium deoxycholate, 1.0% Triton X-100). For SDS-PAGE, 30 µg total protein per sample per lane were loaded, run and transferred to a PVDF membrane, as previously described (Seaberg et al., 2015). Blots were imaged on an Odyssey CLx (LI-COR, Lincoln, NE, USA), and 4× protein sample loading buffer (LI-COR, 928-40004), Chameleon Duo Pre-stained protein ladder (LI-COR, 928-60000) and low-background PVDF membrane (LI-COR, 926-31097) were used as per LI-COR recommendations. Manufacturer instructions and reagents were followed for all steps post-transfer, including Revert 700 Total Protein Staining and Reversing (LI-COR, 926-11010) prior to blocking and probing. Other reagents used were Intercept (TBS) Blocking Buffer (LI-COR, 927-60001) and Intercept T20 (TBS) Antibody Diluent (LI-COR, 927-65001). Primary antibodies were anti-Lyve1 (1:500; Abcam, ab14917, RRID: AB_301509) and anti-Pdpn (1:800; R&D Systems, AF3244, RRID:AB_2268062). Near-infrared secondary antibodies were IRDye 680RD Donkey anti-Goat IgG (H+L) (1:15,000; LI-COR, 926-68074, RRID:AB_10956736) for Pdpn and IRDye 800CW Goat anti-Rabbit IgG (H+L) (1:15,000; LI-COR, 926-32211, RRID:AB_621843) for Lyve1. Blot images were captured and analyzed with LI-COR Image Studio Software v5.5. Loading differences were equalized by dividing the intensity of the antibody band by the lane intensity of the total protein for each sample. Results were normalized relative to the NC controls (set to 1).

### Lymphatic vessel staining and capillary density analysis

Hindlimb muscles were collected and prepared for cryostat sectioning as previously described (Paez-Colasante et al., 2013), except that 2% paraformaldehyde was used for fixation. Cryoprotected tissues were placed in plastic molds, covered in Tissue Tek (OCT, Sakura, Torrence, CA, USA), then frozen and stored at −80°C until use. Cross-sections (14 µm) of EDL from control and ALS animals were cut at −20°C on a cryostat and frozen in serial sections on glass slides that were then stored at −80°C until use. Longitudinal sections (40 µm) of TA from control and ALS animals were cut and frozen as above. Slides were removed from the −80°C freezer and brought up to room temperature (RT) on a benchtop, then moved to a Coplin jar. They were washed 2× in PBS, blocked for 30 min in 125 mM glycine in PBS plus 0.1% Triton X-100 (PBS-T), then washed 2× in PBS. Cross-section slides were blocked for 1 h in a humid chamber with 5% normal goat serum (NGS)+3% BSA in PBS-T and probed overnight with anti-Lyve1 (1:100; Abcam, ab14917, RRID: AB_301509) at 4°C in a humid chamber, while longitudinal slides were blocked with 10% normal donkey serum (NDS) in PBS-T and co-probed overnight with anti-Lyve1 (1:200) and anti-Pdpn (1:200; R&D Systems, AF3244, RRID:AB_2268062). Slides were washed 3× in PBS, then cross-sections were probed for 1 h with Rhodamine Goat anti-Rabbit (1:400; Jackson ImmunoResearch, 111-025-144) at RT in a humid chamber. Longitudinal sections were co-probed with Alexa Fluor 555 Donkey anti-Rabbit (1:1000; Thermo Fisher Scientific, A32794, RRID:AB_2762834) and Alexa Fluor 647 Donkey anti-Goat (1:1000; Thermo Fisher Scientific, A32849, RRID:AB_2762840). Slides were washed 3× in PBS, then mounted with Fluoroshield Mounting Medium with DAPI (Abcam, ab104139) and stored at 4°C in the dark until imaging. For longitudinal sections of TA co-stained for Lyve1 and Pdpn, *z*-stack images of their entire thickness of the TA longitudinal sections (0.75 µm *z*-slice thickness) were taken on an Olympus Fluoview FV3000 confocal microscope with a 20× dry objective, and maximal-intensity projections were generated.

For quantification of lymphatic capillary density on EDL cross sections, *z*-stack images of Lyve1 staining (568 nm) through the entire thickness of the sections (2.0 µm *z*-slice thickness) were taken on the same confocal microscope with a 20× dry objective set to a 2.5× zoom. Images were captured using FV Acquisition FV31S-SW software using a MATL (multiarea time lapse) protocol to create mosaic stitching of overlapping images to form one composite image per section. Five sections were imaged per muscle. These .oir files were converted to .oib files and analyzed with QuPath (v0.4.3) (Bankhead et al., 2017). To measure Lyve1 signal density (Fig. 7C), the polygon tool was used to manually draw an ROI around the whole section in each composite image, then analyzed using the Pixel Classification plugin. The threshold was manually adjusted. By thresholding only the Lyve1 signal points, we obtained the total area of Lyve1 staining. Using the same ROI, the threshold was changed to include the background fluorescence of the cells, giving us the total area of the section. The Lyve1/section area ratios for each section were averaged by muscle, and these values were averaged per genotype. To measure the vessel profile area (Fig. 7D), the same ROI above was analyzed using the Cell Detection plugin. The threshold was again manually adjusted to encompass only the Lyve1 signal, giving us the individual area of Lyve1^+^ profile. These values were averaged for each section, then each section was averaged by muscle, and those values averaged per genotype.

### Tissue clearing

The lumbar spinal column and EDL muscles were dissected following transcardial perfusion with 4% paraformaldehyde and post-fixed overnight at 4°C. Samples were washed with PBS, and the lumbar SPC was dissected from the spinal column. Samples were then processed using a modified iDISCO protocol (Jalufka et al., 2022). Briefly, samples were dehydrated in a series of methanol/water solutions (20%, 40%, 60%, 80%; v/v) for 1 h each, followed by two washes in 100% methanol for 1 h each. Samples were then incubated in 66% dichloromethane (DCM) in methanol overnight at RT. Samples were washed twice with 100% methanol for 30 min each, then rehydrated with the inverse order of the methanol/water solution series used for dehydration. Samples were then washed with PBS for 30 min then incubated in PBS+2% (v/v) Tween 20+10 mg/ml heparin+0.02% (w/v) sodium azide (PTwH) for 30 min. Samples were incubated in PTwH+5% dimethyl sulfoxide (DMSO), 3% NDS+rabbit anti-Lyve1 antibody (1:500; Abcam, ab14917, RRID: AB_301509) for 2 days with gentle rocking at 37°C. They were then washed four times with PTwH for 30 min each, then left in PTwH overnight. Samples were then incubated with PTwH+5% NDS donkey anti-rabbit Alexa Fluor 488 secondary antibody (1:500; Invitrogen, A21206) for 2 days at 37°C with gentle rocking. They were then washed five times with PTwH for 30 min each and left in the final wash overnight at RT with gentle rocking. The samples were mounted in 2% agarose and dehydrated with the same methanol/water series described above, incubated in 66% DCM in methanol for 3 h, washed twice in 100% DCM for 30 min each, then incubated in ethyl cinnamate for 1-2 days prior to imaging.

### 3D lightsheet imaging and image analysis

Cleared tissue samples in ethyl cinnamate were imaged on a Zeiss Z1 lightsheet microscope with a 5× objective (0.16 NA, 0.6-1× optical zoom), 488 nm and 647 nm excitation lasers (at 5% power), and 505-545 nm bandpass and 660 nm long-pass emission filters. *Z*-stack images (3.51 µm *z*-slice thickness) of Lyve1 staining (488 nm) and background fluorescence (647 nm) were captured through the entire thickness of the tissue for each field of view using Zen software (Zeiss). Multiple fields of view were stitched together using Imaris Stitcher to generate a single composite image of the entire tissue. The stitched 3D images were exported as TIFF files from Imaris Software (Bitplane) and analyzed in MATLAB. Owing to the high autofluorescence in muscle tissue, the background channel (647 nm) was subtracted from the Lyve1 staining channel (488 nm) to assess the Lyve1 signal. Following background subtraction, Lyve1 signal was thresholded, and the total number of Lyve1^+^ voxels was counted and divided by the total number of voxels in each tissue.

### Statistical analysis

Statistical analysis was performed with GraphPad Prism. Numerical data for these experiments are expressed as mean±s.e.m. The Shapiro–Wilk test was used to examine data for normality. Normally distributed datasets were compared using two-sample *t*-tests. Non-normally distributed datasets were tested using Mann–Whitney test for unpaired data. *P*<0.05 was considered significant. Figure legends contain sample sizes and statistical tests used for data analysis of each experiment.

## Supplementary Material

10.1242/dmm.052148_sup1 Supplementary information
