# Supplementary material for: Lymphatic dysfunction correlates with inflammation in a mouse model of amyotrophic lateral sclerosis
Source: Dis Model Mech. 2025 Jul 16;18(7):dmm052148. doi: 10.1242/dmm.052148 (PMC12309905; doi:10.1242/dmm.052148)
Supplement: Supplementary information [file dmm-18-052148-s1.pdf]

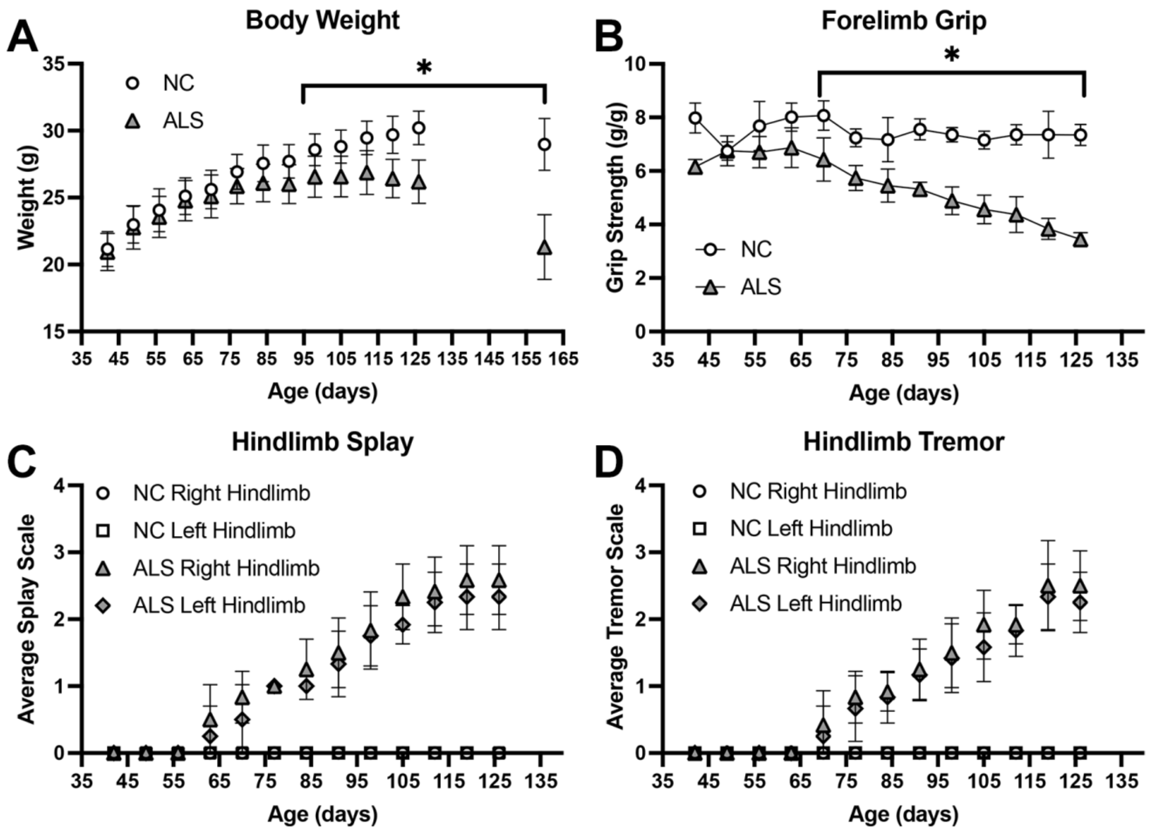

Fig. S1. Phenotyping of SOD1-G93A mice bred locally in our animal facility. All data are for males, presented as mean ± SD. NC=non-carrier control. **(A)** Body weight (g). *N*=12 NC, 12 ALS. Average end-stage age for ALS: 157 ± 11 d; *n*=5 NC, 7 ALS. (*) Mixed-effects analysis, $P<0.05$ through $P<0.0001$.**(B)** Forelimb grip strength normalized to body weight (g/g). (Seaberg et al., 2015). *N*= 6 NC, 6 ALS. (*) Repeated Measures 2-way ANOVA, $P<0.05$ thru $P<0.0001$. **(C)** Hindlimb Splay independently per limb. Scale: 0 normal, 1 mild defect, 2 moderate, 3 strong, 4 paralysis. (Mead et al., 2011). *N*=12 NC, 12 ALS. Scale limb data was averaged per genotype. **(D)** Hindlimb Tremor independently per limb. Scale: 0 normal, 1 mild, 2 moderate, 3 strong tremor (Mead et al., 2011). *N*=12 NC, 12 ALS. Scale limb data was averaged per genotype.

Disease Models & Mechanisms • Supplementary information

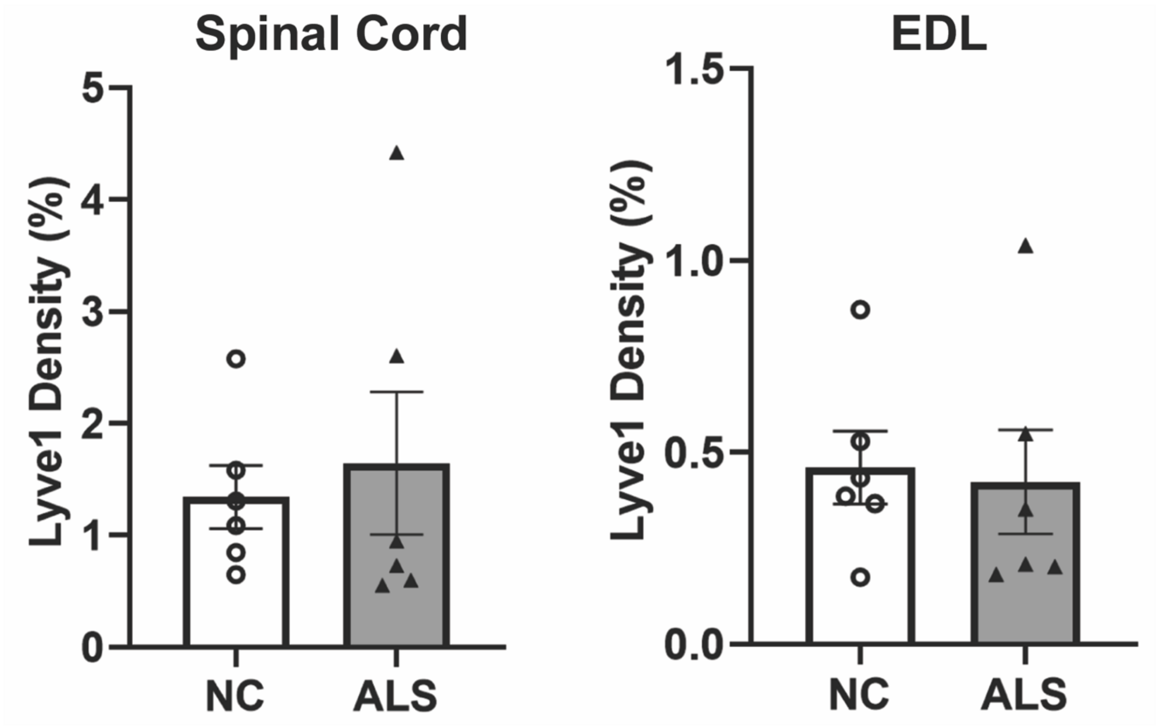

Fig. S2. Lyve1 density in thresholded lightsheet images from p126 female NC and ALS lumbar spinal cord and EDL. N=6/genotype; p=0.69, spinal cord; p=0.69, EDL; unpaired Mann-Whitney test.

Disease Models & Mechanisms • Supplementary information

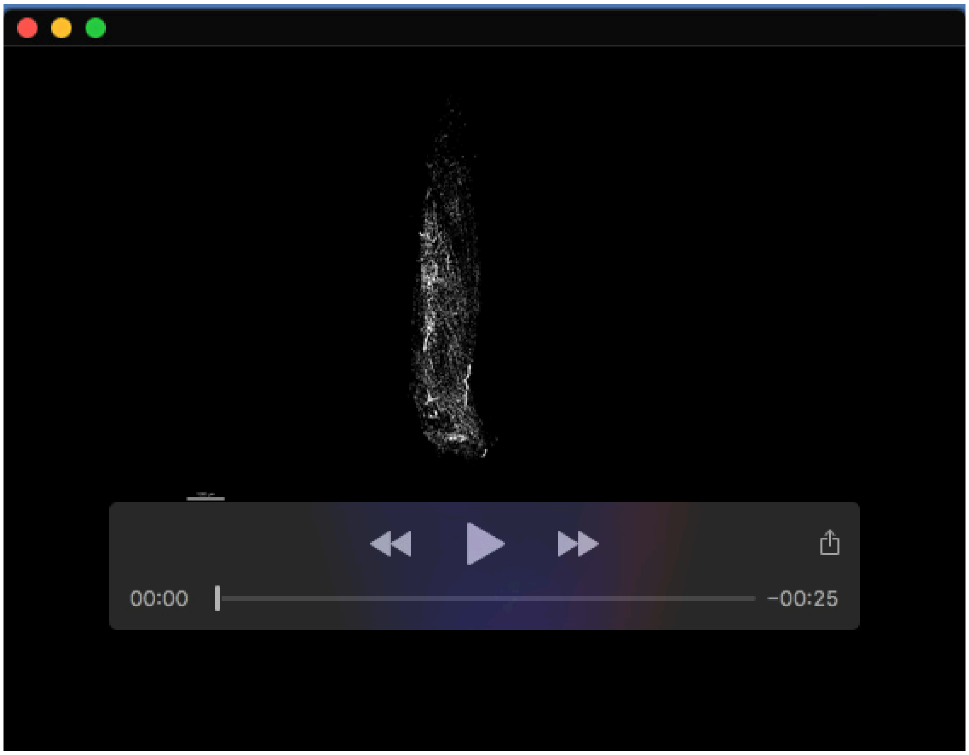

Movie 1. 3D video of thresholded Lyve1 signal through 1 male NC p126 EDL. Scale bar at bottom left.

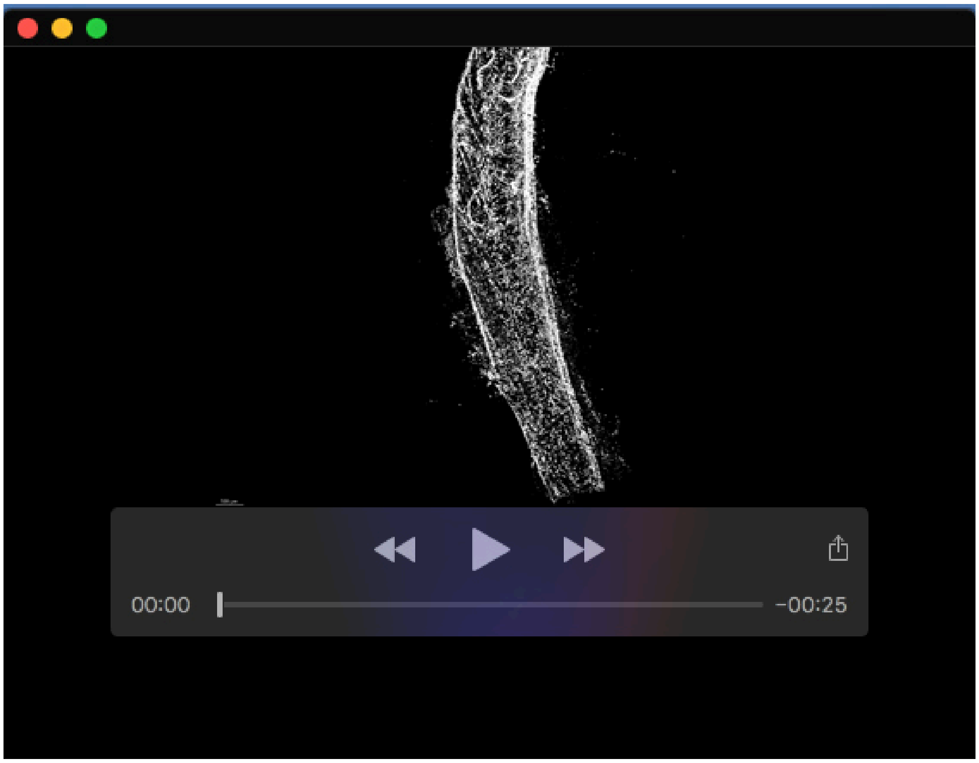

Movie 2. 3D video of thresholded Lyve1 signal through 1 male NC p126 lumbar SC. Scale bar at bottom left.

Disease Models & Mechanisms • Supplementary information

REFERENCES

Mead, R. J., Bennett, E. J., Kennerley, A. J., Sharp, P., Sunyach, C., Kasher, P., Berwick, J., Pettmann, B., Battaglia, G., Azzouz, M., et al. (2011). Optimised and rapid pre-clinical screening in the SOD1(G93A) transgenic mouse model of amyotrophic lateral sclerosis (ALS). *PLoS One*, **6,** e23244.

Seaberg, B., Henslee, G., Wang, S., Paez-Colasante, X., Landreth, G. E. & Rimer, M. (2015). Muscle-derived extracellular signal-regulated kinases 1 and 2 are required for the maintenance of adult myofibers and their neuromuscular junctions. *Molecular and Cellular Biology*, **35,** 1238-1253.

Disease Models & Mechanisms • Supplementary information
